# Supplementary figures and images for: Profiling RT-LAMP tolerance of sequence variation for SARS-CoV-2 RNA detection
Source: PLoS One. 2022 Mar 24;17(3):e0259610. doi: 10.1371/journal.pone.0259610 (PMC8947081; doi:10.1371/journal.pone.0259610)

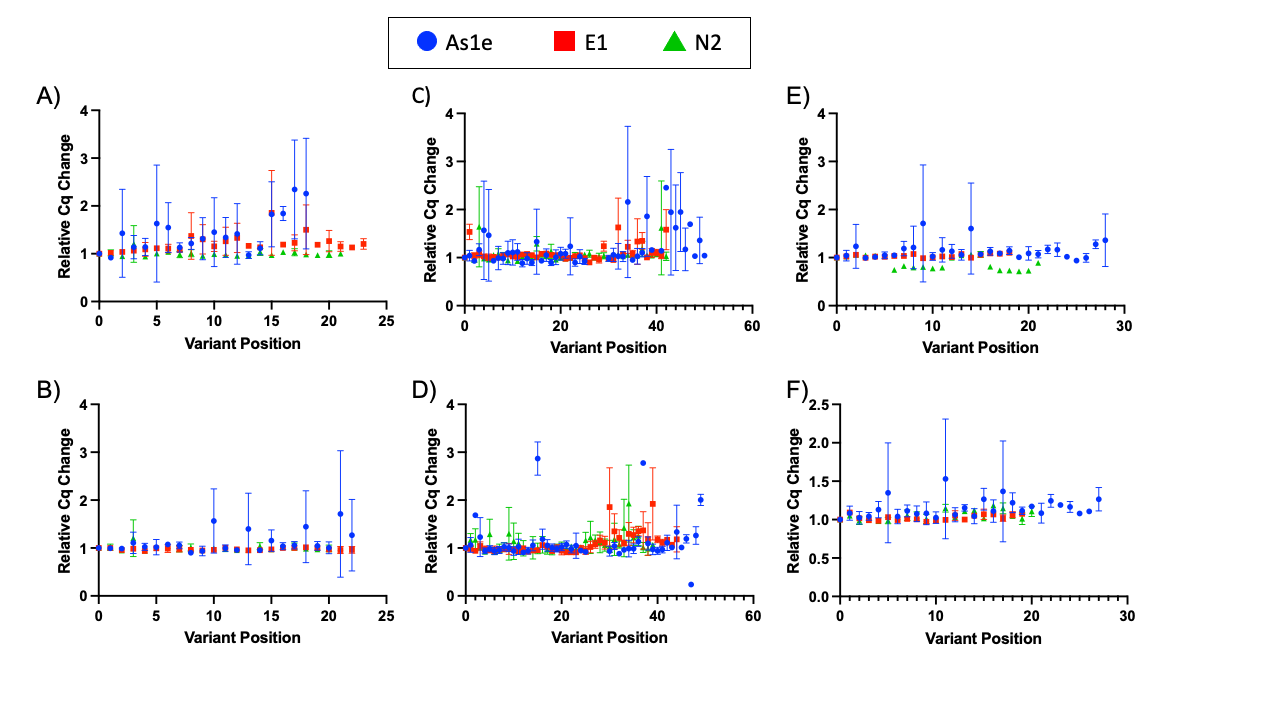

Supplement: S1 Fig — Plots of the effects of change relative to the WT primer set for all three assays explored, As1e (blue circle), E1 (red square), N2 (green triangle) at 100 copies of SARS-CoV-2 RNA. (A) F3 primer, (B) B3 primer, (C) FIP primer, (D) BIP primer, (E) Loop F primer, (F) Loop B primer. (TIFF) [file pone.0259610.s001.tiff]

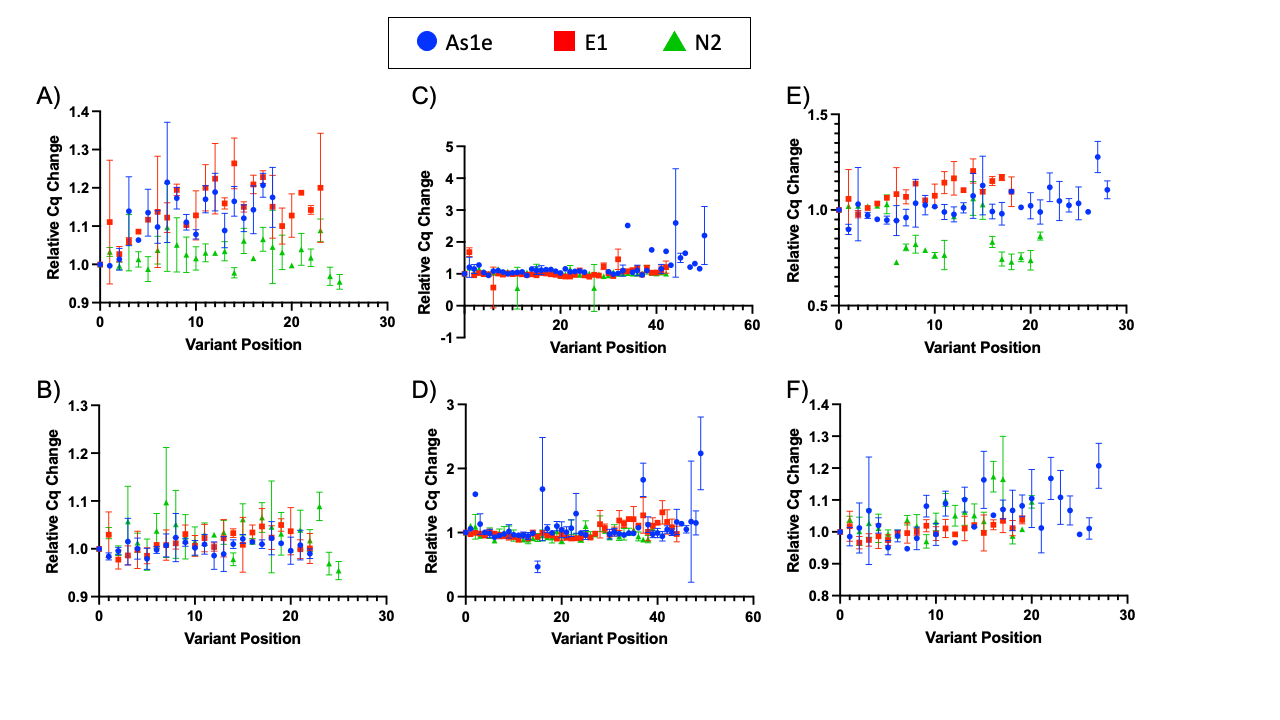

Supplement: S2 Fig — Plots of the effects of change relative to the WT primer set for all three assays explored, As1e (blue circle), E1 (red square), N2 (green triangle) at 200 copies of SARS-CoV-2 RNA. (A) F3 primer, (B) B3 primer, (C) FIP primer, (D) BIP primer, (E) Loop F primer, (F) Loop B primer. (TIFF) [file pone.0259610.s002.tiff]

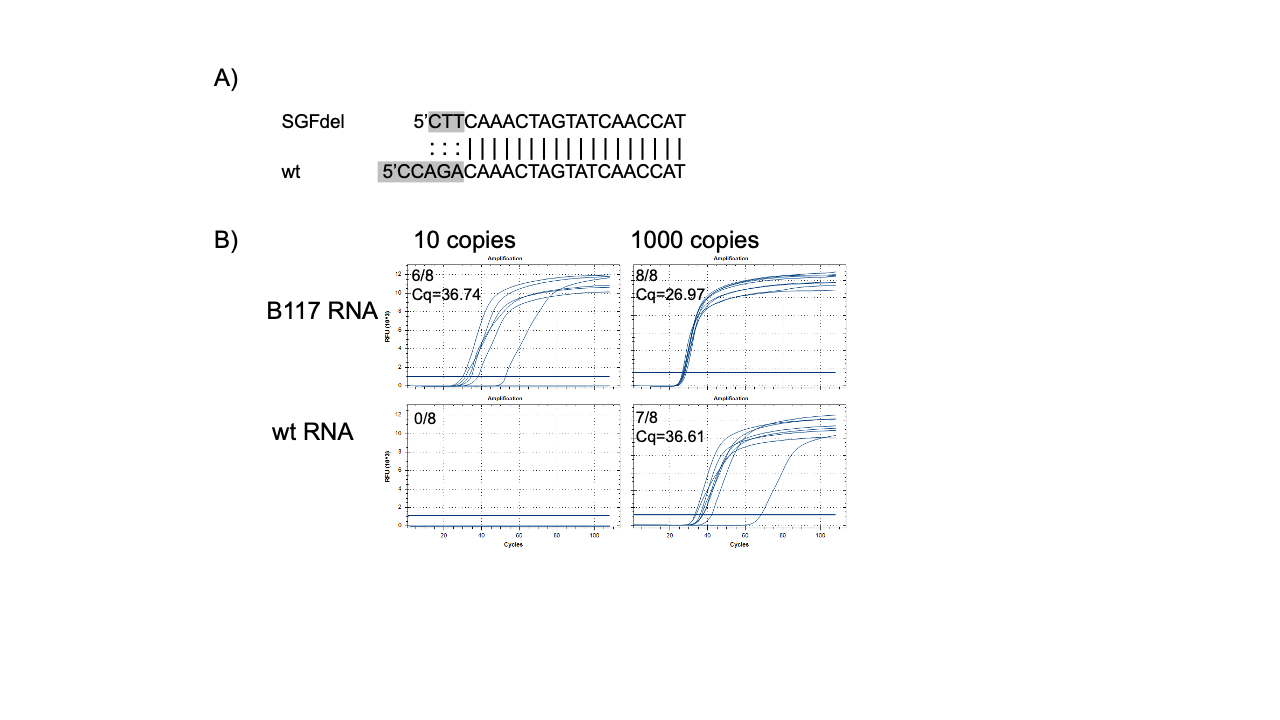

Supplement: S3 Fig — (A) Comparison of sequences of the 5’ of the FIP primers for detecting wt or SGF deletion. (B) LAMP amplification with primer set containing SGFdel-FIP. 8x repeats of LAMP reactions were performed with 1000 or 10 copies of RNA from B117 or wt. The speed of LAMP is shown as “# of Cq” with each Cq equal to 24s. Positive amplification threshold is Cq<60. (TIFF) [file pone.0259610.s003.tiff]
